# Relationships between Neonatal Weight, Limb Lengths, Skinfold Thicknesses, Body Breadths and Circumferences in an Australian Cohort

**DOI:** 10.1371/journal.pone.0105108

**Published:** 2014-08-27

**Authors:** Emma Pomeroy, Jay T. Stock, Tim J. Cole, Michael O'Callaghan, Jonathan C. K. Wells

**Affiliations:** 1 Newnham College, University of Cambridge, Cambridge, United Kingdom; 2 Division of Biological Anthropology, Department of Archaeology and Anthropology, University of Cambridge, Cambridge, United Kingdom; 3 Population Policy and Practice, UCL Institute of Child Health, London, United Kingdom; 4 School of Medicine, Mater Clinical School, University of Queensland, Brisbane, Queensland, Australia; 5 Childhood Nutrition Research Centre, UCL Institute of Child Health, London, United Kingdom; The Ohio State Unversity, United States of America

## Abstract

**Background:**

Low birth weight has been consistently associated with adult chronic disease risk. The thrifty phenotype hypothesis assumes that reduced fetal growth impacts some organs more than others. However, it remains unclear how birth weight relates to different body components, such as circumferences, adiposity, body segment lengths and limb proportions. We hypothesized that these components vary in their relationship to birth weight.

**Methods:**

We analysed the relationship between birth weight and detailed anthropometry in 1270 singleton live-born neonates (668 male) from the Mater-University of Queensland Study of Pregnancy (Brisbane, Australia). We tested adjusted anthropometry for correlations with birth weight. We then performed stepwise multiple regression on birth weight of: body lengths, breadths and circumferences; relative limb to neck-rump proportions; or skinfold thicknesses. All analyses were adjusted for sex and gestational age, and used logged data.

**Results:**

Circumferences, especially chest, were most strongly related to birth weight, while segment lengths (neck-rump, thigh, upper arm, and especially lower arm and lower leg) were relatively weakly related to birth weight, and limb lengths relative to neck-rump length showed no relationship. Skinfolds accounted for 36% of birth weight variance, but adjusting for size (neck-rump, thigh and upper arm lengths, and head circumference), this decreased to 10%. There was no evidence that heavier babies had proportionally thicker skinfolds.

**Conclusions:**

Neonatal body measurements vary in their association with birth weight: head and chest circumferences showed the strongest associations while limb segment lengths did not relate strongly to birth weight. After adjusting for body size, subcutaneous fatness accounted for a smaller proportion of birth weight variance than previously reported. While heavier babies had absolutely thicker skinfolds, this was proportional to their size. Relative limb to trunk length was unrelated to birth weight, suggesting that limb proportions at birth do not index factors relevant to prenatal life.

## Introduction

Neonatal characteristics such as birth weight, ponderal index, or relative length and head circumference may be considered proxies for prenatal environmental quality and are associated with the risk of developing various non-communicable diseases (NCDs, e.g. type 2 diabetes, cardiovascular disease) in later life [1]–[2]. These associations formed the basis of the thrifty phenotype hypothesis [3], which has proved highly influential in theorising the cause of relationships between early life conditions, growth, and the risk of NCDs in adulthood. Under adverse environmental conditions, the body appears to prioritise growth in certain organs such as the brain at the expense of others such as the pancreas, heart, liver, kidneys and skeletal muscle [3]–[5]. These trade-offs may have negative consequences in later life, particularly where compromised metabolic function resulting from poor early growth is exposed to a westernised lifestyle (rich diet and reduced activity) [3]. Poor early growth combined with an obesogenic adult environment may be particularly problematic in low-middle income countries where the risk of low birth weight remains high and transitions to westernised lifestyles are occurring rapidly [6]–[9].

Many studies concerned with the thrifty phenotype hypothesis use the same basic indicators of prenatal growth (birth weight, ponderal index, relative head circumference), but the relationship between birth weight and other neonatal anthropometry is not well characterised.

For example, the relative contribution of adiposity to variation in birth weight is uncertain. One often-cited study reported that total fat mass of neonates explained 46% of the variance in birth weight even though adipose comprises only around 12% of total birth weight [10], but whether heavier babies are also proportionally fatter remains unclear. Relative fatness, rather than absolute fat mass, is likely to be a stronger influence on later disease risk and be a more relevant indicator of neonatal nutritional status [11]–[15]. Understanding the relationship between birth weight and a wider range of neonatal anthropometric characteristics, including limb and trunk lengths, skinfolds, body breadths and circumferences, may offer novel insight into variation in the proportionality of prenatal growth across the birth weight spectrum and into prenatal growth trade-offs in the context of environmental adaptation or accommodation.

It is also unclear how limb lengths and proportions relate to birth weight. This may be relevant as shorter limbs relative to trunk length in adulthood are associated with elevated NCD risk (e.g. [16]–[23]) and postnatal limb proportions may be particularly sensitive to environmental stressors [24]–[31]. Studies investigating the effects of specific stressors on neonatal development (maternal smoking [32]–[33] or diabetes [12], [34]) suggest that neonatal trunk, limb and limb segment lengths and proportions are indeed differentially affected. Lampl et al [32] reported a relatively shorter tibia compared to the thigh, and lower limb relative to upper limb length, among maternal smoke-exposed mid-gestation foetuses, while Lindsay et al. [33] reported that tibia and forearm lengths, and especially thigh length, showed greater differences between control and maternal smoke-exposed neonates than total crown-rump length. In relation to diabetes, Lampl et al. [34] found stronger effects in the lower limb than the upper limb and in the tibia than the femur, while Catalano et al. [12] reported that upper arm, lower arm and lower leg lengths, but not crown-rump or thigh lengths, were significantly smaller in diabetes-exposed neonates, though differences were modest. However, broader trends in these characteristics relative to birth weight are unknown.

The purpose of this study is to investigate the relationship between birth weight and detailed anthropometric measurements in a large sample (n = 1270) of neonates from Brisbane, Australia, including skinfold measurements, limb segment and trunk lengths, and body breadths and circumferences. We aimed to understand the proportionality between birth weight and these different neonatal measurements, and test the hypotheses based on existing literature that shorter segment lengths, especially smaller skinfolds and absolute and relative limb lengths, are associated with lower birth weight.

## Materials and Methods

Data on neonatal birth weight, anthropometry, gestational age and sex from the Mater-University of Queensland Study of Pregnancy (MUSP) [35] were analysed. As part of a larger study (n = 7223 neonates), detailed anthropometry was collected on 1272 neonates (live singleton births, 668 male) born between June 1982 and September 1983 in Brisbane, Australia [36]. Exclusion criteria for this phase of the study were: multiple pregnancy; congenital abnormalities; baby admitted to intensive neonatal care or unstable medically; and mothers whose dates were quite uncertain (since routine ultrasounds were not performed in the early 1980s). This should be considered a convenience sample, since study staff endeavoured to see as many newborns as possible but were not able to capture all eligible births. However, no specific selection criteria were applied, and the infants for whom detailed anthropometry were available did not differ significantly from other participants in the study in either birth weight or sex ratio, but gestation was very slightly longer in our sample compared with the full cohort (39.7 vs. 39.8 weeks respectively) [36].

The following measurements were recorded: birth weight; neck-rump, upper arm, forearm, thigh, and lower leg lengths; head, chest, abdominal, upper arm (MUAC), lower arm, thigh and lower leg circumferences; biparietal, face, shoulder and hip width; and subscapular, triceps, abdominal and anterior thigh skinfold thicknesses. All measurements were taken by the same trained research nurse following standard techniques [36]–[37]. Two individuals were excluded from analyses due to erroneous measurements or multiple congenital birth defects, leaving a sample of 1270. A small number of measurements were missing, so sample sizes for individual analyses are given as appropriate. Normality of data distributions was evaluated using histograms prior to analyses.

As birth weight (which relates to volume) may scale allometrically with the other anthropometry (linear measurements), natural logarithms of all anthropometry were used in analyses, although results changed little whether log transformed or raw data were used. The relationship between different neonatal measurements was first assessed using Pearson's correlation. Correlations were performed for the sexes separately, adjusting for gestational age, and for the sexes combined, adjusting for gestational age and sex. Multiple regression of each measurement on birth weight, adjusting for gestation and sex and including a sex*anthropometry interaction term indicated no sex differences in the relationships between birth weight and the various measurements (results not shown). The sexes were therefore pooled for subsequent analyses.

To further investigate the relationship between birth weight and neonatal anthropometry, gestational age- and sex-adjusted standardised residuals were first derived for the variables using multiple regression, given evidence for differences in birth weight, body composition and body size/proportions along these lines [38]–[41]. Stepwise multiple regression of anthropometry residuals on birth weight residual was then performed. Variables were entered into the model when p<0.05, and excluded where p>0.10. Body breadths, lengths and circumferences were included in the first analysis, while skinfolds were considered in a separate analysis, since body circumferences and skinfold thicknesses capture some of the same variation in body size and composition. As absolute skinfold thicknesses may simply increase proportionally with greater neonatal size, analyses were conducted on unadjusted skinfold measurements and also adjusting for measurements that reflect the overall size or size of the appropriate segments where skinfolds were measured (neck-rump, upper arm and thigh lengths, and head circumference) to investigate whether skinfold thicknesses scaled allometrically or isometrically with birth weight.

To analyse the relationship between birth weight and relative limb proportions, a gestational age- and sex-adjusted ratio of upper or lower limb length to trunk length (calculated as (proximal+distal limb segment lengths)/neck-rump length) was regressed on the standardised residual for birth weight, with and without adjusting for head circumference (to reflect overall size).

Finally, reduced major axis (RMA) regression of the sum of 4 skinfold thicknesses or each of the individual skinfolds on birth weight was performed, adjusting for sex and gestational age, to assess whether skinfold thicknesses scale proportionally with weight. RMA regression was selected as the dependent-independent relationship between the variables is unclear, allometric relationships were under investigation, and the purpose of the analysis was not to derive predictive equations [42]. Analyses were conducted using SPSS for Windows version 21.0

### Ethics statement

The ethics committees of the Mater Hospitals and the University of Queensland approved the study. Oral informed consent was obtained at recruitment to the study, as approved by the ethics committees and in line with standards for human research at the time (early 1980s). Informed consent was documented on a specific form for this purpose.

## Results

Mean (standard deviation) birth weight in the sample was 3446 (450) g, and mean gestational age was 39.7 (1.3) weeks. Summary statistics for all raw anthropometry are given in Table 1.

**Table 1:** Summary statistics for the study sample (raw data, not log transformed).

Characteristic	Female (n = 602)		Male (n = 668)		Total (n = 1270)	
	Mean	SD	Mean	SD	Mean	SD
Gestation (weeks)	39.7	1.2	39.7	1.3	39.7	1.3
Birth weight (g)	3381	453	3504	438	3446	450
Head circumference (mm)	348.0	11.75	354.1	12.29	351.2	12.41
Biparietal diameter (mm)	93.7	3.52	95.0	3.65	94.4	3.64
Face diameter (mm)	85.6	4.15	86.9	4.30	86.3	4.28
Neck-rump length (mm)	226.6	14.47	228.5	14.82	227.6	14.68
Shoulders width (mm)	156.5	10.13	158.3	10.56	157.5	10.39
Hips width (mm)	132.4	10.46	133.7	10.71	133.1	10.61
Upper arm length (mm)	83.0	6.60	84.7^a^	6.89	83.9	6.80
Mid upper arm circumference (mm)	109.2	9.22	110.0	9.06	109.6	9.14
Lower arm length (mm)	60.2^a^	8.15	61.7	8.00	61.0	8.10
Lower arm circumference (mm)	99.7^a^	7.75	100.6	7.30	100.2	7.52
Chest circumference (mm)	332.6	16.84	334.9	17.01	333.8	16.96
Abdomen circumference (mm)	288.3^b^	19.83	287.6	18.99	287.9	19.38
Thigh length (mm)	89.2^c^	6.78	90.2	6.70	89.7	6.76
Thigh circumference (mm)	154.9	13.71	153.7	13.13	154.3	13.41
Lower leg length (mm)	68.1	7.93	69.6	8.12	68.9	8.06
Lower leg circumference (mm)	112.3	8.64	112.9	8.42	112.6	8.53
Subscapular skinfold (mm)	54.9	10.43	52.6	10.61	53.7	10.58
Abdominal skinfold (mm)	35.3	6.07	35.4	6.51	35.4	6.31
Triceps skinfold (mm)	49.6	9.26	48.9	8.90	49.2	9.08
Anterior thigh skinfold (mm)	66.9	13.97	62.8	13.63	64.7	13.93

a = 1 missing data point.

b = 2 missing data points.

c = 3 missing data points.

**Figure 1:**
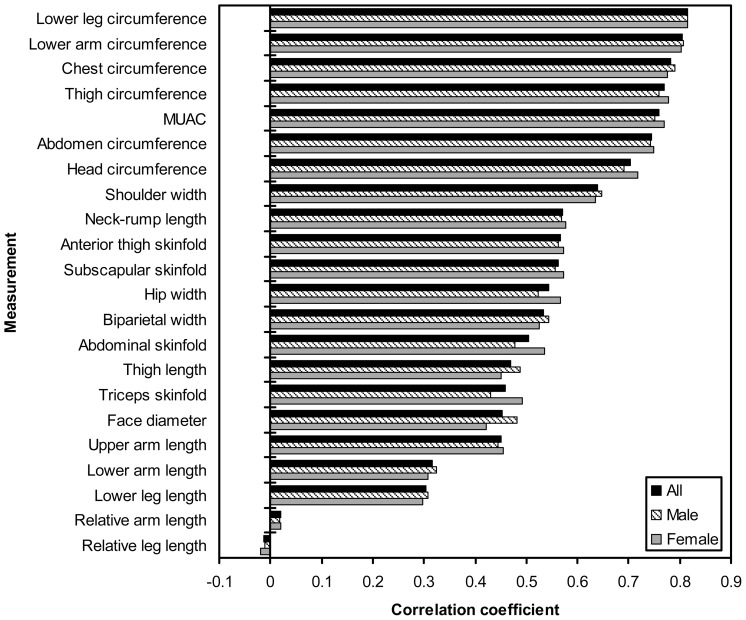
Correlations between birth weight and neonatal body measurements. Adjusted for gestational age for males and females separately, and for gestational age and sex for combined sexes (‘All’). Anthropometry log transformed prior to analysis. MUAC = mid upper arm circumference.

Correlations between birth weight and anthropometry (adjusting for gestational age) show minor differences between the sexes (Figure 1). Pooling the sexes and adjusting for gestational age and sex, body circumferences showed the highest correlation with birth weight, ranging from 0.70 (head) to 0.82 (lower leg). Body breadths and neck-rump length showed the next highest correlations (r ranging from 0.45–0.64), which were similar to those for skinfolds (r = 0.46 to 0.57). Limb segment lengths, especially distal segment lengths, showed the lowest correlations with birth weight (r = 0.30 to 0.47). In general, limb segment lengths and particularly distal limb segment lengths showed the weakest correlations with other measurements (Table S1), including with neck-rump length (lower arm: r = 0.15, lower leg r = 0.16) and particularly weak relationships with body breadths (r = −0.21 to 0.28).

**Figure 2:**
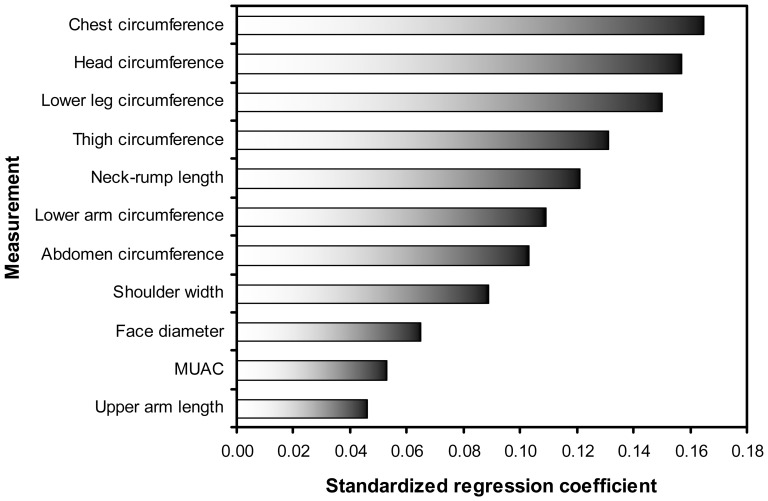
Regression coefficients for stepwise multiple regression of birth weight on body lengths, breadths and circumferences. Anthropometry log transformed prior to analysis. MUAC = mid upper arm circumference.

The stepwise multiple regression analysis highlighted a similar pattern of relationships between birth weight and body lengths and circumferences (Figure 2, Table S2). Neonatal anthropometry explained 88% of the variance in birth weight adjusting for sex and gestation. Lower leg, head, chest and thigh circumferences were the first four variables to enter the model, followed by neck-rump length, and then shoulder width, abdominal and lower arm circumferences, face diameter, upper arm length and finally MUAC. Biparietal and hip widths, and thigh, lower arm and lower leg lengths were excluded by the stepwise procedure. In the final model, regression coefficients were strongest for chest and head circumferences and lowest for upper arm length and MUAC (Figure 2).

**Figure 3:**
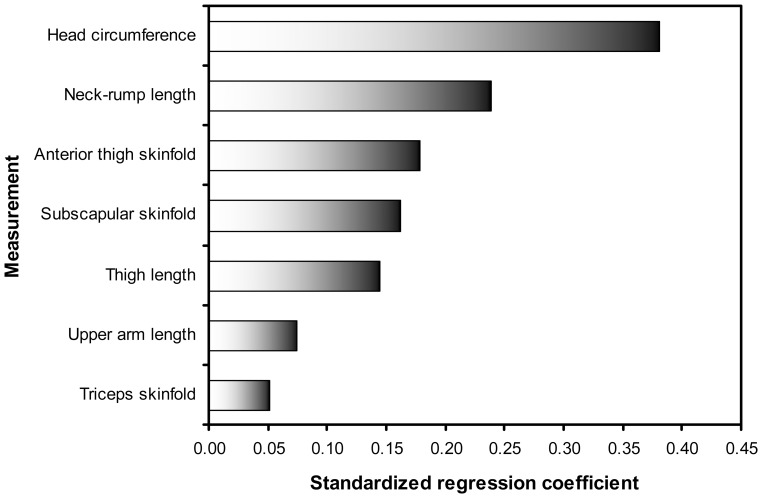
Regression coefficients for stepwise multiple regression of birth weight on skinfold thicknesses, adjusting for overall size. Anthropometry log transformed prior to analysis.

The multiple regression of birth weight on the four skinfold measurements showed a significant relationship between each of the skinfolds and birth weight (p<0.001, adjusted R^2^ attributable to skinfolds = 0.36). However, adjusted for indicators of overall size (head circumference and neck-rump, upper arm and thigh lengths), abdominal skinfold was no longer significant in the regression model (Figure 3, Table S3), and the contribution of skinfolds to explaining variance in birth weight was more modest (adjusted R^2^ value attributable to skinfolds = 0.10, compared with overall adjusted R^2^ of 0.77; model p<0.001).

Relative lower and upper limb lengths showed no significant relationship with birth weight, adjusting for gestational age and sex (p = 0.6 and 0.5 respectively), a result that did not change when head circumference was added to the model (p = 0.7 and 0.4 respectively; details not shown).

**Figure 4:**
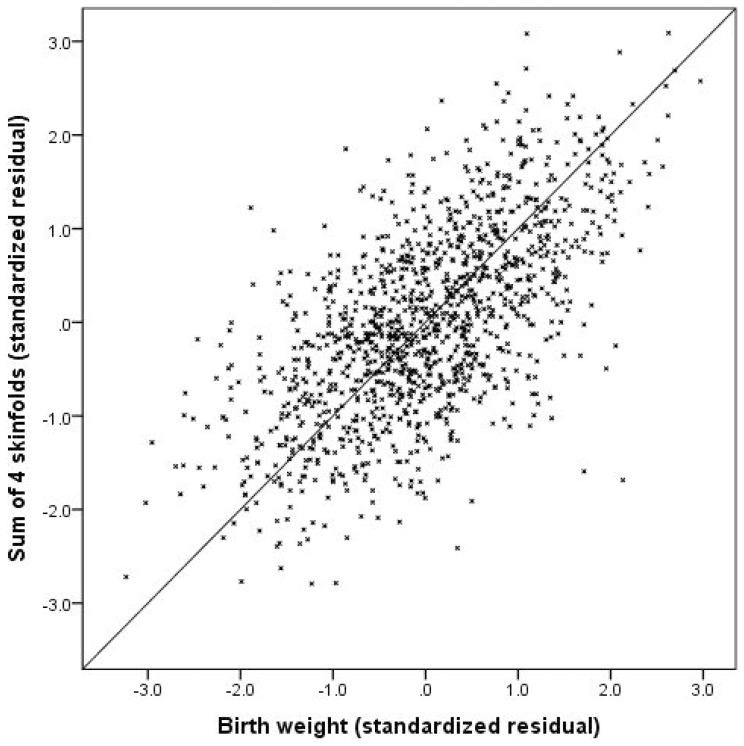
Scatterplot of sum of 4 skinfolds against birth weight. Data are standardised residuals from regression analysis to adjust log transformed anthropometry for sex and gestational age.

The RMA regression slope of the sum of the 4 skinfolds on birth weight (adjusted for both sex and gestational age) was 1.00, indicating isometry in the relationship between skinfold thickness and birth weight (Figure 4). Plots of individual skinfold residuals against those for birth weight follow a very similar pattern (not shown).

## Discussion

Our study shows that neonatal anthropometric traits vary in the strength of their associations with birth weight. Body circumferences were most strongly related to birth weight and while heavier babies had absolutely larger skinfolds, this relationship was markedly attenuated once we accounted for overall size. Limb segment lengths showed weak associations with birth weight, and relative limb to trunk lengths showed no relationship to birth weight.

The fact that body circumferences, particularly those of the chest and head, show the strongest relationships with birth weight is perhaps unsurprising. The trunk and head form, by volume and weight, the greatest part of the neonatal body [43], while limbs are relatively underdeveloped at birth and experience accelerated postnatal growth relative to the head and trunk [26], [44]. Furthermore, trunk and limb circumferences summarise the amount of both lean and fat tissue, so along with body length, may be expected to be major determinants of birth weight. It is also unsurprising therefore that neck-rump length is next most strongly associated with birth weight after body circumferences. A number of previous studies have shown that of body circumferences, chest circumference is among the measurements most strongly related to birth weight [43], [45]–[50]. Typical correlation coefficients of 0.7 are reported and it has been argued that chest circumference may be a useful proxy for low birth weight in resource-poor settings [47]–[48].

Our results support a previous study showing that heavier babies have greater fat mass (represented here by skinfolds), and that neonatal fat mass explains approximately 46% of the variance in birth weight [10]. In our data, 36% of variance in birth weight was explained by the four skinfold measurements adjusting for sex and gestation. While Catalano et al. estimated fat mass for their analyses from skinfolds, using conversion equations to estimate fat mass increases the associated errors [51]. As skinfold thicknesses are proportional to fat mass, our use of skinfolds adjusted for gestation and sex offer a comparable measure to that used by Catalano et al. [10].

Our support for Catalano et al.' s findings comes, however, with a caveat: our results also further highlight the potential misconception that fat mass alone explains this proportion of the birth weight variance. Numerous studies cite Catalano et al.'s result that while fat constitutes 12% of neonatal mass, it accounts for 46% of the variance in birth weight [52]–[58], and some state that fat mass explains more variation in birth weight than does lean mass [59]. However, this is only recognising part of this relationship. Babies who are larger overall could be expected to have greater lean and fat mass, and once major correlates of lean mass that are minimally influenced by fat mass are accounted for (namely head circumference and neck-rump, thigh and upper arm lengths), the variance in birth weight explained by skinfolds fell to 10% in our dataset. This too is consistent with Catalano et al.'s [10] results, since they pointed out that estimated lean mass accounted for 85% of the variance in birth weight, and recognised that with the variance accounted for by fat mass exceeded 100%, since the two components are unlikely to be entirely independent. However, our data suggest that fat mass may not be as useful per se as an indicator of prenatal growth, since adiposity (represented by subcutaneous fat, or sum of 4 skinfolds) relative to birth weight shows a similar degree of variability across the birth weight range

The relatively weak associations between limb length measurements and birth weight, as well as other anthropometry including body breadths and neck-rump length, suggest a considerable degree of independence between limb segment lengths and other neonatal dimensions. The lack of association between relative limb to trunk lengths and birth weight is consistent with previous arguments that relative lower limb length indexes postnatal, not prenatal, environment [60]–[62]. These previous studies examined associations between relative limb proportions in childhood or adulthood with birth weight, so their results could also be explained if neonatal limb proportions are associated with prenatal environment, but this relationship is subsequently erased by postnatal growth. However, our study indicates that relative limb lengths at birth are not associated with birth weight, an overall proxy for prenatal environmental quality. Another previous study (albeit using a small sample) also suggested that small for gestational age babies show little difference in limb proportions from those born appropriate gestational age [63]. Our findings therefore support the interpretation of limb proportions as markers of postnatal environment.

The results, however, contrast with several studies that report reduced relative limb or limb segment lengths due to exposure to specific prenatal stressors such as maternal smoking [32]–[33] or diabetes [12], [34], despite the fact that birth weight is often increased in cases of maternal diabetes [11]–[12], [14]. Lampl and colleagues have argued that both maternal smoking and diabetes cause foetal hypoxia [64], accounting for their similar effects on relative limb lengths. If this is the case, it may be that only certain prenatal exposures influence limb proportions, while relative limb lengths and proportions act as a more general indicator of environmental stress exposure postnatally. At present, the mechanisms by which hypoxia and other environmental stressors affect relative limb growth both pre- and postnatally are unknown, thus the potential for differences in these mechanisms according to phase of development that would be needed to support this model cannot currently be assessed.

It is particularly relevant that distal limb segment lengths show weak associations with birth weight and other anthropometry. Distal limb segment length is argued to be especially sensitive to postnatal environment compared with total limb length in the lower [26], [28], [30], [65], and upper [28]–[29] limbs. Future research should aim to assess the link between prenatal environment and relative limb and limb segment proportions both at birth and in later life, and examine the differential contributions of both pre- and postnatal environments to patterns of adult disease risk. Considering the evidence for both pre- and postnatal life, it appears that associations between birth size, limb proportions and risk derive from different growth periods. Both prenatal and postnatal environment may have separate influences on chronic disease risk in adulthood, since both birth weight and relative limb proportions have been related to chronic disease risk, and support a model of cumulative disease risk through the life course [66]. Relative limb proportions may therefore be useful for investigating the contribution of postnatal vs. prenatal environmental factors for the accumulation of risk during infancy and childhood.

Body composition, rather than birth weight, is likely to be a more relevant means of assessing neonatal nutritional status. This is particularly important given that populations differ systematically in body composition. For example, South Asian infants of a given birth weight have a similar fat mass but reduced lean mass compared with Western populations (the so-called thin-fat phenotype) [15], [67], so birth weight may not adequately reflect either nutritional status or later disease risk. Furthermore, infants born to mothers with obesity and/or gestational diabetes are at risk of having a higher proportion of body fat at birth, but do not necessarily have greater total birth weight [11]–[14], [68]. Therefore birth weight alone may not identify those at risk of adverse health consequences associated with higher neonatal fat mass, including excess adiposity and obesity risk in childhood [69]–[70]. Neonatal nutritional status may be best assessed by relative lean and fat mass proportions, rather than fat mass or birth weight [13], [15], [71]. However, much of the literature relates NCD risk to birth weight or ponderal index rather than neonatal body composition, and thus studies are needed to assess the link specifically between neonatal body composition and NCD risk in later life.

A limitation of this study is that it did not directly assess the relationship between anthropometry and markers of prenatal environment. Birth weight has limitations as a marker of foetal environmental stress exposure, and assessing relative limb proportions in relation to other prenatal environment indicators may be more appropriate for assessing the use of limb proportions as markers of prenatal environment. Furthermore, skinfolds measure only subcutaneous fat, and do not consider deeper fat deposits, which could relate differently to birth weight. Finally, this relationship between relative limb proportions, subcutaneous fat and birth weight may not apply in other populations with different environmental exposures or ancestry, and this needs to be investigated. For example, one study showed that heavier neonates from Bangalore, India, had proportionally thicker skinfolds (and so greater percentage fat mass) than normal or low birth weight infants [15].

While the maternally-reported ethnicity of the parents in the sample was overwhelmingly ‘White’ (91% of 1216 mothers and 93% of 1167 fathers on whom data were available, remaining parents split approximately equally between ‘Asian’ and ‘Aboriginal/Islander’), body size, composition and proportions throughout life are known to be affected by ancestry [67], [72]–[74]. Ancestry may have therefore influenced the results, although given the preponderance of ‘White’ ethnicity these influences are likely to have been minor, and no genetic data were available for either parents or offspring to enable us to assess fully the potential impact of ancestry. However, future work should consider how the relationship between neonatal size, body proportions and skinfold thicknesses vary among populations.

Finally, we note that this sample comprises infants born in 1982–3 in a small region of Australia. Thus it is uncertain whether the relationships reported here between different neonatal measurements remain the same in contemporary birth cohorts, or babies from other regions. Nevertheless the results provide an important first insight into the relationships between different aspects of neonatal anthropometry, and question which few datasets have the required anthropometric detail to address.

In conclusion, our study shows that different body measurements at birth show varying relationships to overall birth weight. Head and chest circumferences show the closest associations with birth weight, while relative limb proportions at birth are unrelated to birth weight and therefore do not appear to act as markers of prenatal environment. The results also suggest that subcutaneous fat explains less of the variation in birth weight than previously argued. Rather, in our sample, subcutaneous fat appears to increase proportionally with birth weight. This finding supports arguments that body composition, rather than birth weight or fat mass, may be more appropriate for assessing newborn nutritional status.

## Supporting Information

Table S1 **Correlation between anthropometry (adjusted for gestational age and sex).**
(DOC)

Table S2 **Results of stepwise multiple regression of anthropometric variables on birth weight (adjusting for gestational age and sex, n = 1263).** Variables listed in the order they entered the model in the stepwise procedure. Variables excluded from the model (p>0.10): biparietal and hip widths, lower arm length, thigh and lower leg lengths. SE = standard error. Anthropometry log transformed prior to analysis. MUAC = mid upper arm circumference.
(DOC)

Table S3 **Results of multiple regression of birth weight on skinfolds and body size (head circumference and neck-rump, thigh and upper arm lengths, n = 1266).** Variable excluded from the model (p>0.10): abdominal skinfold. SE = standard error. Anthropometry log transformed prior to analysis.
(DOC)
