# Supplementary material for: Relationships between Neonatal Weight, Limb Lengths, Skinfold Thicknesses, Body Breadths and Circumferences in an Australian Cohort
Source: PLoS One. 2014 Aug 27;9(8):e105108. doi: 10.1371/journal.pone.0105108 (PMC4146506; doi:10.1371/journal.pone.0105108)
Supplement: Table S1 — Correlation between anthropometry (adjusted for gestational age and sex). (DOC) [file pone.0105108.s001.doc]

**Table S1. Correlation between anthropometry (adjusted for gestational age and sex).**

| **Measurements** | | **Birth weight** | **Lengths** | | | | | **Widths** | | | | **Circumferences** | | | | | | | **Skinfolds** | | | | **Proportions** | |
| --- | --- | --- | --- | --- | --- | --- | --- | --- | --- | --- | --- | --- | --- | --- | --- | --- | --- | --- | --- | --- | --- | --- | --- | --- |
|  |  |  | **Neck-rump** | **Upper arm** | **Lower arm** | **Thigh** | **Lower leg** | **Bi-parietal** | **Face** | **Shoulder** | **Hip** | **Head** | **Chest** | **Abdomen** | **MUAC** | **Lower arm** | **Thigh** | **Lower leg** | **Sub-scapular** | **Triceps** | **Abdomen** | **Anterior thigh** | **RUL** | **RLL** |
| **Birth weight** | |  | **0.57** | **0.45** | **0.32** | **0.47** | **0.30** | **0.53** | **0.45** | **0.64** | **0.54** | **0.70** | **0.78** | **0.75** | **0.76** | **0.80** | **0.77** | **0.82** | **0.56** | **0.46** | **0.50** | **0.57** | 0.02 | -0.01 |
| **Lengths** | **Neck-rump** | **0.57** |  | **0.25** | **0.15** | **0.32** | **0.16** | **0.30** | **0.31** | **0.38** | **0.36** | **0.42** | **0.46** | **0.42** | **0.34** | **0.43** | **0.42** | **0.44** | **0.22** | **0.24** | **0.25** | **0.25** | **-0.43** | **-0.45** |
|  | **Upper arm** | **0.45** | **0.25** |  | **0.71** | **0.73** | **0.66** | **0.18** | -0.04 | **0.28** | **0.19** | **0.33** | **0.44** | **0.46** | **0.46** | **0.40** | **0.32** | **0.39** | **0.22** | ***0.07*** | **0.37** | **0.19** | **0.69** | **0.53** |
|  | **Lower arm** | **0.32** | **0.15** | **0.71** |  | **0.65** | **0.79** | 0.05 | **-0.21** | **0.11** | 0.01 | **0.22** | **0.35** | **0.40** | **0.36** | **0.31** | **0.24** | **0.28** | **0.12** | -0.01 | **0.33** | 0.04 | **0.78** | **0.63** |
|  | **Thigh** | **0.47** | **0.32** | **0.73** | **0.65** |  | **0.69** | **0.17** | -0.03 | **0.22** | **0.11** | **0.36** | **0.51** | **0.48** | **0.40** | **0.40** | **0.33** | **0.41** | **0.19** | **0.*06*** | **0.36** | **0.08** | **0.49** | **0.61** |
|  | **Lower leg** | **0.30** | **0.16** | **0.66** | **0.79** | **0.69** |  | 0.05 | **-0.19** | ***0.07*** | -0.04 | **0.22** | **0.37** | **0.39** | **0.30** | **0.28** | **0.22** | **0.27** | ***0.06*** | -0.01 | **0.31** | -0.02 | **0.63** | **0.75** |
| **Widths** | **Biparietal** | **0.53** | **0.30** | **0.18** | 0.05 | **0.17** | 0.05 |  | **0.70** | **0.42** | **0.39** | **0.67** | **0.41** | **0.36** | **0.42** | **0.40** | **0.40** | **0.40** | **0.25** | **0.22** | **0.16** | **0.31** | **-0.08** | **-0.11** |
|  | **Face** | **0.45** | **0.31** | -0.04 | **-0.21** | -0.03 | **-0.19** | **0.70** |  | **0.45** | **0.49** | **0.50** | **0.29** | **0.25** | **0.33** | **0.32** | **0.36** | **0.34** | **0.24** | **0.26** | 0.06 | **0.37** | **-0.33** | **-0.33** |
|  | **Shoulder** | **0.64** | **0.38** | **0.28** | **0.11** | **0.22** | ***0.07*** | **0.42** | **0.45** |  | **0.71** | **0.47** | **0.50** | **0.47** | **0.55** | **0.54** | **0.55** | **0.56** | **0.39** | **0.33** | **0.30** | **0.48** | -0.05 | **-0.12** |
|  | **Hip** | **0.54** | **0.36** | **0.19** | 0.01 | **0.11** | -0.04 | **0.39** | **0.49** | **0.71** |  | **0.39** | **0.36** | **0.39** | **0.50** | **0.46** | **0.48** | **0.48** | **0.38** | **0.32** | **0.21** | **0.53** | **-0.13** | **-0.22** |
| **Circumferences** | **Head** | **0.70** | **0.42** | **0.33** | **0.22** | **0.36** | **0.22** | **0.67** | **0.50** | **0.47** | **0.39** |  | **0.58** | **0.54** | **0.56** | **0.58** | **0.51** | **0.56** | **0.35** | **0.31** | **0.33** | **0.38** | 0.01 | -0.01 |
|  | **Chest** | **0.78** | **0.46** | **0.44** | **0.35** | **0.51** | **0.37** | **0.41** | **0.29** | **0.50** | **0.36** | **0.58** |  | **0.71** | **0.65** | **0.69** | **0.64** | **0.70** | **0.47** | **0.35** | **0.46** | **0.41** | **0.11** | **0.12** |
|  | **Abdomen** | **0.75** | **0.42** | **0.46** | **0.40** | **0.48** | **0.39** | **0.36** | **0.25** | **0.47** | **0.39** | **0.54** | **0.71** |  | **0.66** | **0.68** | **0.64** | **0.68** | **0.50** | **0.37** | **0.48** | **0.47** | **0.17** | **0.14** |
|  | **MUAC** | **0.76** | **0.34** | **0.46** | **0.36** | **0.40** | **0.30** | **0.42** | **0.33** | **0.55** | **0.50** | **0.56** | **0.65** | **0.66** |  | **0.81** | **0.70** | **0.77** | **0.58** | **0.42** | **0.49** | **0.60** | **0.20** | **0.11** |
|  | **Lower arm** | **0.80** | **0.43** | **0.40** | **0.31** | **0.40** | **0.28** | **0.40** | **0.32** | **0.54** | **0.46** | **0.58** | **0.69** | **0.68** | **0.81** |  | **0.74** | **0.82** | **0.59** | **0.46** | **0.53** | **0.55** | **0.09** | 0.04 |
|  | **Thigh** | **0.77** | **0.42** | **0.32** | **0.24** | **0.33** | **0.22** | **0.40** | **0.36** | **0.55** | **0.48** | **0.51** | **0.64** | **0.64** | **0.70** | **0.74** |  | **0.76** | **0.53** | **0.44** | **0.46** | **0.58** | 0.01 | -0.02 |
|  | **Lower leg** | **0.82** | **0.44** | **0.39** | **0.28** | **0.41** | **0.27** | **0.40** | **0.34** | **0.56** | **0.48** | **0.56** | **0.70** | **0.68** | **0.77** | **0.82** | **0.76** |  | **0.60** | **0.47** | **0.54** | **0.57** | 0.05 | 0.03 |
| **Skinfolds** | **Subscapular** | **0.56** | **0.22** | **0.22** | **0.12** | **0.19** | ***0.06*** | **0.25** | **0.24** | **0.39** | **0.38** | **0.35** | **0.47** | **0.50** | **0.58** | **0.59** | **0.53** | **0.60** |  | **0.60** | **0.58** | **0.69** | 0.02 | -0.03 |
|  | **Triceps** | **0.46** | **0.24** | ***0.07*** | -0.01 | **0.*06*** | -0.01 | **0.22** | **0.26** | **0.33** | **0.32** | **0.31** | **0.35** | **0.37** | **0.42** | **0.46** | **0.44** | **0.47** | **0.60** |  | **0.44** | **0.61** | **-0.13** | **-0.14** |
|  | **Abdomen** | **0.50** | **0.25** | **0.37** | **0.33** | **0.36** | **0.31** | **0.16** | 0.06 | **0.30** | **0.21** | **0.33** | **0.46** | **0.48** | **0.49** | **0.53** | **0.46** | **0.54** | **0.58** | **0.44** |  | **0.51** | **0.19** | **0.16** |
|  | **Anterior thigh** | **0.57** | **0.25** | **0.19** | 0.04 | **0.08** | -0.02 | **0.31** | **0.37** | **0.48** | **0.53** | **0.38** | **0.41** | **0.47** | **0.60** | **0.55** | **0.58** | **0.57** | **0.69** | **0.61** | **0.51** |  | -0.05 | **-0.15** |
| **Proportion** | **RUL** | 0.02 | **-0.43** | **0.69** | **0.78** | **0.49** | **0.63** | **-0.08** | **-0.33** | -0.05 | **-0.13** | 0.01 | **0.11** | **0.17** | **0.20** | **0.09** | 0.01 | 0.05 | 0.02 | **-0.13** | **0.19** | -0.05 |  | **0.86** |
|  | **RLL** | -0.01 | **-0.45** | **0.53** | **0.63** | **0.61** | **0.75** | **-0.11** | **-0.33** | **-0.12** | **-0.22** | -0.01 | **0.12** | **0.14** | **0.11** | 0.04 | -0.02 | 0.03 | -0.03 | **-0.14** | **0.16** | **-0.15** | **0.86** |  |

**Bold** indicates p<0.01, ***bold italic*** indicates p<0.05. Anthropometry log transformed prior to analysis. MUAC = mid upper arm circumference. RUL = relative upper limb length; RLL = relative lower limb length.
