# Supplementary material for: Relationships between Neonatal Weight, Limb Lengths, Skinfold Thicknesses, Body Breadths and Circumferences in an Australian Cohort
Source: PLoS One. 2014 Aug 27;9(8):e105108. doi: 10.1371/journal.pone.0105108 (PMC4146506; doi:10.1371/journal.pone.0105108)
Supplement: Table S2 — Results of stepwise multiple regression of anthropometric variables on birth weight (adjusting for gestational age and sex, n = 1263). Variables listed in the order they entered the model in the stepwise procedure. Variables excluded from the model (p>0.10): biparietal and hip widths, lower arm length, thigh and lower leg lengths. SE = standard error. Anthropometry log transformed prior to analysis. MUAC = mid upper arm circumference. (DOC) [file pone.0105108.s002.doc]

**Table S2. Results of stepwise multiple regression of anthropometric variables on birth weight (adjusting for gestational age and sex, n=1263).**

| **Model term** | **b** | **SE (b)** | **β** | **p** |
| --- | --- | --- | --- | --- |
| (Constant) | -5.75 | 0.25 | *-* | <0.001 |
| Sex | 0.01 | 0.003 | 0.05 | <0.001 |
| Gestation | 0.001 | 0.001 | 0.01 | 0.3 |
| Lower leg circumference | 0.26 | 0.04 | 0.15 | <0.001 |
| Chest circumference | 0.43 | 0.04 | 0.17 | <0.001 |
| Head circumference | 0.58 | 0.06 | 0.16 | <0.001 |
| Thigh circumference | 0.20 | 0.03 | 0.13 | <0.001 |
| Neck-rump length | 0.25 | 0.03 | 0.12 | <0.001 |
| Shoulder width | 0.18 | 0.03 | 0.09 | <0.001 |
| Abdomen circumference | 0.20 | 0.03 | 0.10 | <0.001 |
| Lower arm circumference | 0.19 | 0.04 | 0.11 | <0.001 |
| Face diameter | 0.17 | 0.03 | 0.07 | <0.001 |
| Upper arm length | 0.07 | 0.02 | 0.05 | <0.001 |
| MUAC | 0.08 | 0.03 | 0.05 | 0.007 |

Variables listed in the order they entered the model in the stepwise procedure .Variables excluded from the model (p>0.10): biparietal and hip widths, lower arm length, thigh and lower leg lengths. SE = standard error. Anthropometry log transformed prior to analysis. MUAC = mid upper arm circumference.
