# Supplementary material for: Relationships between Neonatal Weight, Limb Lengths, Skinfold Thicknesses, Body Breadths and Circumferences in an Australian Cohort
Source: PLoS One. 2014 Aug 27;9(8):e105108. doi: 10.1371/journal.pone.0105108 (PMC4146506; doi:10.1371/journal.pone.0105108)
Supplement: Table S3 — Results of multiple regression of birth weight on skinfolds and body size (head circumference and neck-rump, thigh and upper arm lengths, n = 1266). Variable excluded from the model (p>0.10): abdominal skinfold. SE = standard error. Anthropometry log transformed prior to analysis. (DOC) [file pone.0105108.s003.doc]

**Table S3. Results of multiple regression of birth weight on skinfolds and body size (head circumference and neck-rump, thigh and upper arm lengths, n=1266).**

| **Model term** | **b** | **SE (b)** | **β** | **p** |
| --- | --- | --- | --- | --- |
| (Constant) | -5.73 | 0.32 | - | <0.001 |
| Sex | 0.02 | 0.004 | 0.06 | <0.001 |
| Gestation | 0.01 | 0.002 | 0.06 | <0.001 |
| Head circumference | 1.41 | 0.07 | 0.38 | <0.001 |
| Subscapular skinfold | 0.11 | 0.01 | 0.16 | <0.001 |
| Neck-rump length | 0.49 | 0.03 | 0.24 | <0.001 |
| Thigh length | 0.25 | 0.04 | 0.15 | <0.001 |
| Anterior thigh skinfold | 0.11 | 0.01 | 0.18 | <0.001 |
| Upper arm length | 0.12 | 0.03 | 0.07 | <0.001 |
| Triceps skinfold | 0.04 | 0.01 | 0.05 | 0.006 |

Variable excluded from the model (p>0.10): abdominal skinfold. SE = standard error. Anthropometry log transformed prior to analysis.
